# Biochar and fulvic acid amendments mitigate negative effects of coastal saline soil and improve crop yields in a three year field trial

**DOI:** 10.1038/s41598-020-65730-6

**Published:** 2020-06-02

**Authors:** Yun-peng Sun, Jing-song Yang, Rong-jiang Yao, Xiao-bing Chen, Xiang-ping Wang

**Affiliations:** 1 State Key Laboratory of Soil and Sustainable Agriculture, Institute of Soil Science, Chinese Academy of Sciences, Nanjing, 210008 China; 2 University of Chinese Academy of Sciences, Yuquan Road, Beijing, 100049 China; 3 Yantai Coastal Zone Research Institute, Chinese Academy of Sciences, 264003 Yantai, China

**Keywords:** Agroecology, Natural hazards

## Abstract

China with large area of land planted with crops are suffering secondary salinization in coastal area for the lack of fresh water and saltwater intrusion to the groundwater. The purpose of this study was to investigate the effects of biochar (BC) and fulvic acid (FA) on the amelioration of coastal saline soil and their impact on crop yields under maize-barley rotation system. A three year field experiment was conducted in a saline soil on a farm in coastal area of east Jiangsu Province, China. A maize-barley rotation system had been carried out for ten years with local conventional management before the experiment. The saline soil was amended with BC at rates of 0, 7.5 t ha^−1^ (BC1), 15 t ha^−1^ (BC2) and 30 t ha^−1^ (BC3) alone or combined with fulvic acid (1.5 t ha^−1^) compared with control. Fertilizers were applied under normal planting strategies. The BC was added only once during the four growing seasons, and the FA was applied before each sowing. Soil salinity changed significantly during the three year field experiment. This was mainly due to the great quantity of rain during the period of maize cultivation. Although Na^+^, Cl^−^ and SO_4_^2−^ in BC and /or FA treatments significantly decreased, the pH value increased up to 9.0 as the CO_3_^2−^ + HCO_3_^−^content increased. Total organic carbon (TOC) and phosphorus (TP) responded positively to biochar addition rate. BC applied with appropriate rate at 15 t ha^−1^ (BC2) in combination with FA showed optimal effects on soil salinity amelioration, soil physics properties regulation, soil nutrition improvement and crop yields increase. The TOC and TP was 5.2 g kg^−1^ and 507 mg kg^−1^ in BC2 + FA treatment, which were lower than BC3 and BC3 + FA treatments. However, the highest total grain yield was obtained in the BC2 + FA treatment, and the total yield was increased by 62.9% over the CK. This study emphasizes that using combined organic amendment of BC with FA for profitable and sustainable use of salt-affected soils would be practicable.

## Introduction

Soil salinization has been one of the major environmental problems threatening agricultural productivity since ancient times and is increasing steadily in many parts of the world^1,2^. The total area of saline soil in the world is approximately 831 million hectares, extending over all the continents including Asia, Africa, Australasia, and the Americas^3^. As an important land resource, the coastal saline soil occurs widely in the Eastern China. In the coastal area of Jiangsu Province, eastern China, the reclaimed tidal flat is approximately 2 million hectares, and the land area is gradually increasing at a rate of 1300 hectares every year^4^. The coastal saline soils were developed from highly saline mud flats, and it have been used for agriculture and supply shortages in farm area trigged by increased food production requirements^5^. However, many specific problems such as low nutrient, saline groundwater, accumulation of sodium, scare fresh water, and lower microbial diversity have always been the limiting factors of the coastal saline soil, and eventually restrict the growth of crops^6^. Therefore, in order to improve the productivity of coastal saline soil, management methods need to be developed to improve the soil properties and to decrease soluble salt content of the tillage layer soil.

There are several remediation options for saline sodic soils, which can be grouped into three types: vegetative bioremediation with halophyte^7^, leaching^8^, or addition of either chemical^9^ or organic amendments^10^. However, leaching needs lots of fresh water^11^, and the bioremediation takes a long time^12^, which make it difficult to remediate the saline soil with short time and low cost. Otherwise, the stability of coastal ecosystem is more sensitive than that in inland areas^13^. More attention should paid on the environment protection during the soil amelioration and crops cultivation. Although inorganic materials, such as gypsum, zeolite and bottom ash, have also been reported to ameliorate the saline sodic soil properties, repeated application will result in heavy metal accumulation in the soil^14^.

Application of organic matter to saline sodic soil is considered a good practice for soil remediation^15,16^. Addition of organic materials; such as crop residue, compost, humic acid and biochar have been reported to improve the soil quality of reclaimed tidal land soil^4,16–20^. However, the pathogens remained in plant residues and compost may cause a variety of crop diseases^21,22^, which makes it difficult to take advantage of it for a long time. Besides, this two kinds of materials decomposed quickly in soil^23^, and the character of short-term carbon sequestration would be adverse to climate mitigation.

Biochar (BC), a carbon-rich residue produced under oxygen-limited conditions at temperatures ranging from 300 to 1000 °C, has attracted big attention as a salt-affected soil amendment^24^. Furthermore, the application of BC to soil represents a situation of long-term carbon sequestration^25^. Biochar addition can reduce soil salt stress through sorption^26^, and it can also improving nutrition availability^27,28^.

Fulvic acid (FA), one of two classes of natural acidic organic polymer that can be extracted from humus found in sediment, soil, or aquatic environments, is a fraction of soil organic matter^29^. Moreover, FA is believed to originate as a product of microbial metabolism, and it plays a stimulant role in protection of crop against salt stress^30^, although it is not synthesized as a life-sustaining carbon or energy source^31^.

Though each material has its advantages and disadvantages, the combination of BC and FA may provide improvements than use each of them alone. The stability and absorption properties of BC can offer stable environment for FA and prevent it from loss. Otherwise, the carboxyl groups (COOH) in FA is especially reactive with metals and leading to their increased solubility in soil waters^32^, which is effective for nutrient release of BC.

Even though there are many studies dealing with biochar and humic acid for ameliorating soil salinity, very little is known about their effects on crop growth and soil properties under the rotation system in a long time. Besides, many studies have focused on the soils that had been no crops planted. However, the large areas of land planted with crops are suffering soil salinization. For the lack of effective remediation methods and the urgent need of food, a large area of crops planted in saline soils has lasted for years in China. The aim of this study was to evaluate the effects of biochar and fulvic acid on soil quality and crop performance under maize-barley rotation over a three year period. Although the experimental soil had been cultivated for ten years by native farmers, the crop yields were extremely low. We hypothesized that biochar and fulvic acid substrates will bring positive effects on crop production and soil salt amelioration.

## Results

### Changes in soil salinity

**Figure 1:**
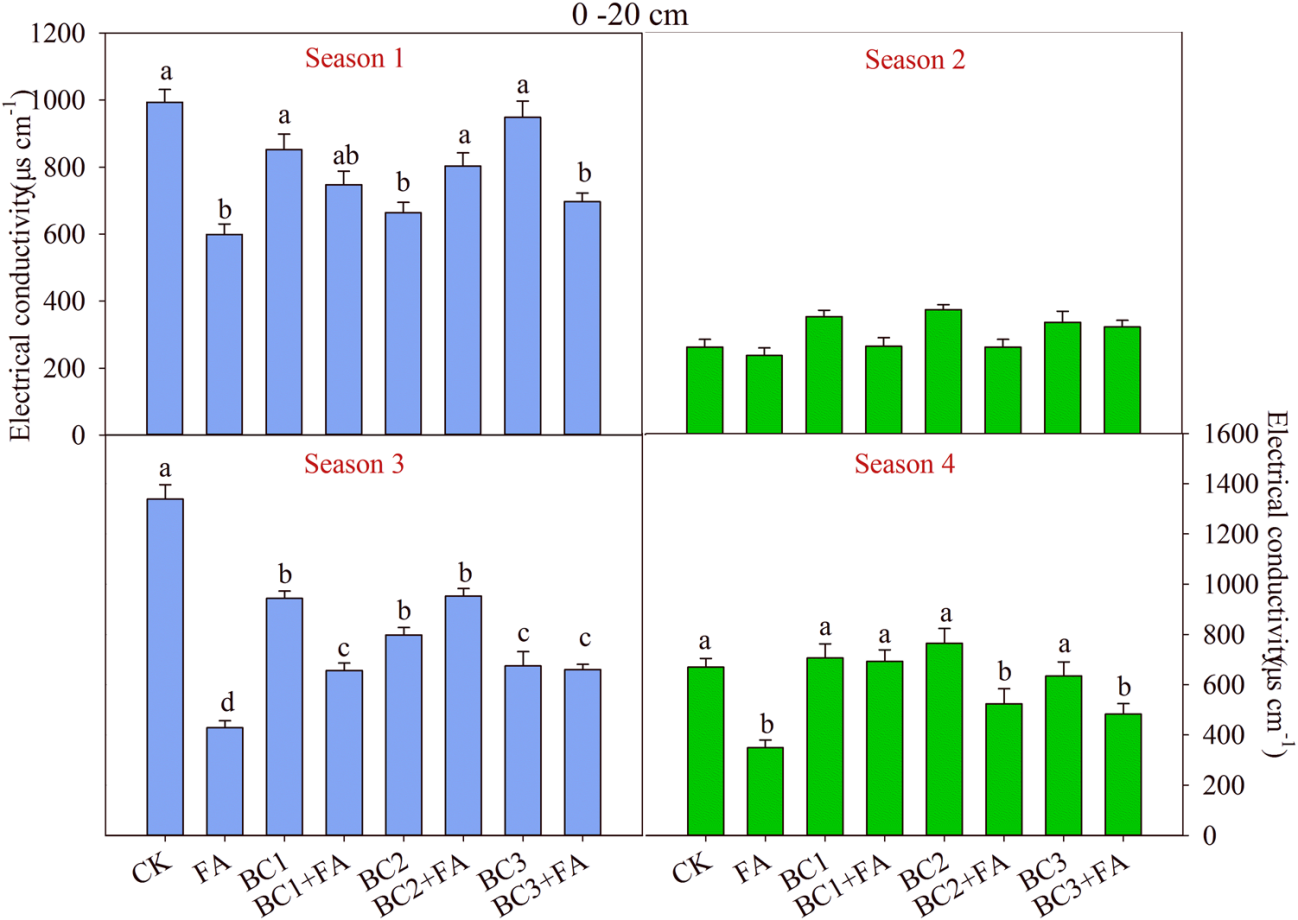
Soil electrical conductivity (EC) at 0–20 cm depth as affected by different treatments during the studied four seasons. CK is control, BC1 is 7.5 t ha^−1^ biochar, BC2 is 15 t ha^−1^ biochar, BC3 is 30 t ha^−1^ biochar, FA is 1.5 t ha^−1^ fulvic acid. Values are means ± SE (*n* = 3). For each season, bars within each panel with different letters are significantly different according to LSD at *p* < 0.05 level. Bars with the same fill color represent the same crop species during the experiment.

**Figure 2:**
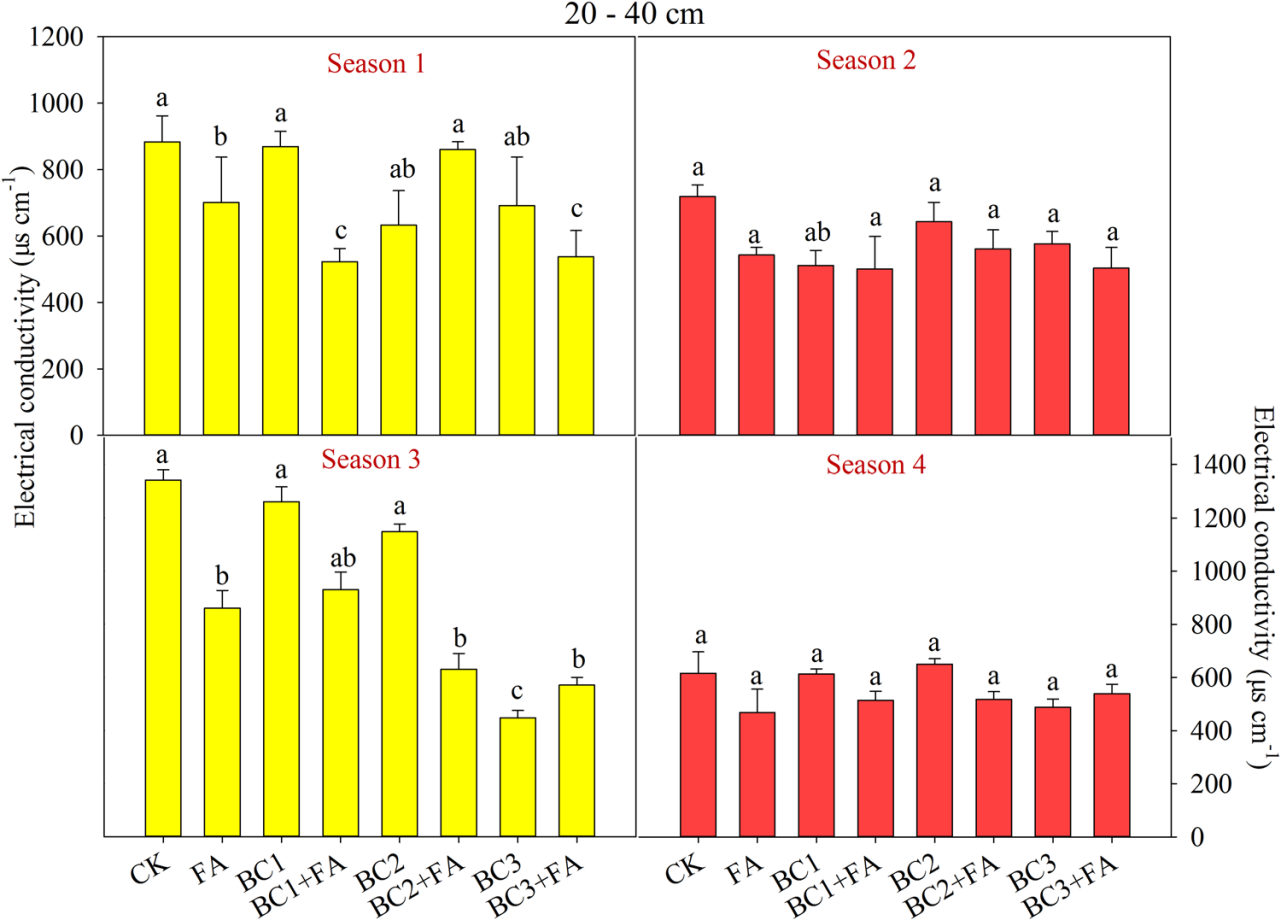
Soil electrical conductivity (EC) at 20–40 cm depth as affected by different treatments during the studied four seasons. CK is control, BC1 is 7.5 t ha^−1^ biochar, BC2 is 15 t ha^−1^ biochar, BC3 is 30 t ha^−1^ biochar, FA is 1.5 t ha^−1^ fulvic acid. Values are means ± SE (*n* = 3). For each season, bars within each panel with different letters are significantly different according to LSD at *p* < 0.05 level. Bars with the same fill color represent the same crop species during the experiment.

Compared with CK, all treatments reduced soil EC in the 0–20 cm soil layer among the four growing seasons (Fig. 1). Only FA, BC2 + FA and BC3 + FA, when compared to CK, decreased EC significantly (*P* < 0.05) at the end of the experiment. This was mainly caused by high variation of rain between growing seasons. And the soil EC of the three treatment was 349, 523 and 482 μs cm^−1^, respectively. Biochar addition didn’t increase soil EC in the season 1, and there were no significant difference between all treatments in the next season. The soil EC in season 1 and 3 were all higher than that in season 2 and 4, which indicates the situation of soil salt accumulated at depth of 0–20 cm during maize cultivation and decreased during barley season. In the 20–40 cm soil layer, there were no significant differences in soil EC after the season 2 and season 4 (*P* > 0.05). Though in general the soil EC was decreased at depth of 20–40 cm in the season 1 and season 3, only FA, BC3 and BC3 + FA treatments decreased EC significantly (*P* < 0.05) compared with CK (Fig. 2). The soil EC of each treatment at 0–20 cm layer in season 1 were higher than that of 20–40 cm, but lower in season 2 and season 4.

**Figure 3:**
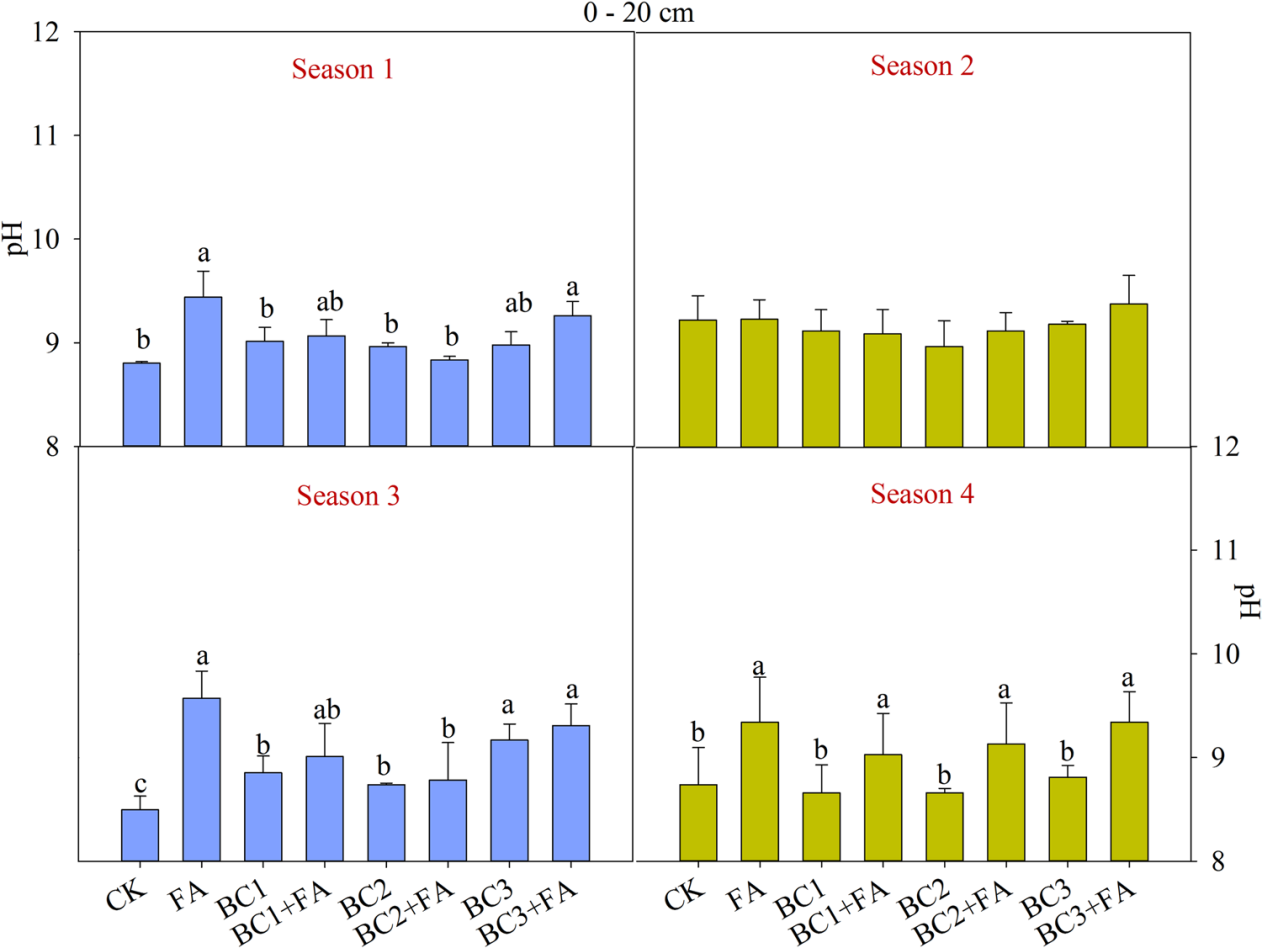
Soil pH values at 0–20 cm depth as affected by different treatments during the studied four seasons. CK is control, BC1 is 7.5 t ha^−1^ biochar, BC2 is 15 t ha^−1^ biochar, BC3 is 30 t ha^−1^ biochar, FA is 1.5 t ha^−1^ fulvic acid. Values are means ± SE (*n* = 3). For each season, bars within each panel with different letters are significantly different according to LSD at *p* < 0.05 level. Bars with the same fill color represent the same crop species during the experiment.

**Figure 4:**
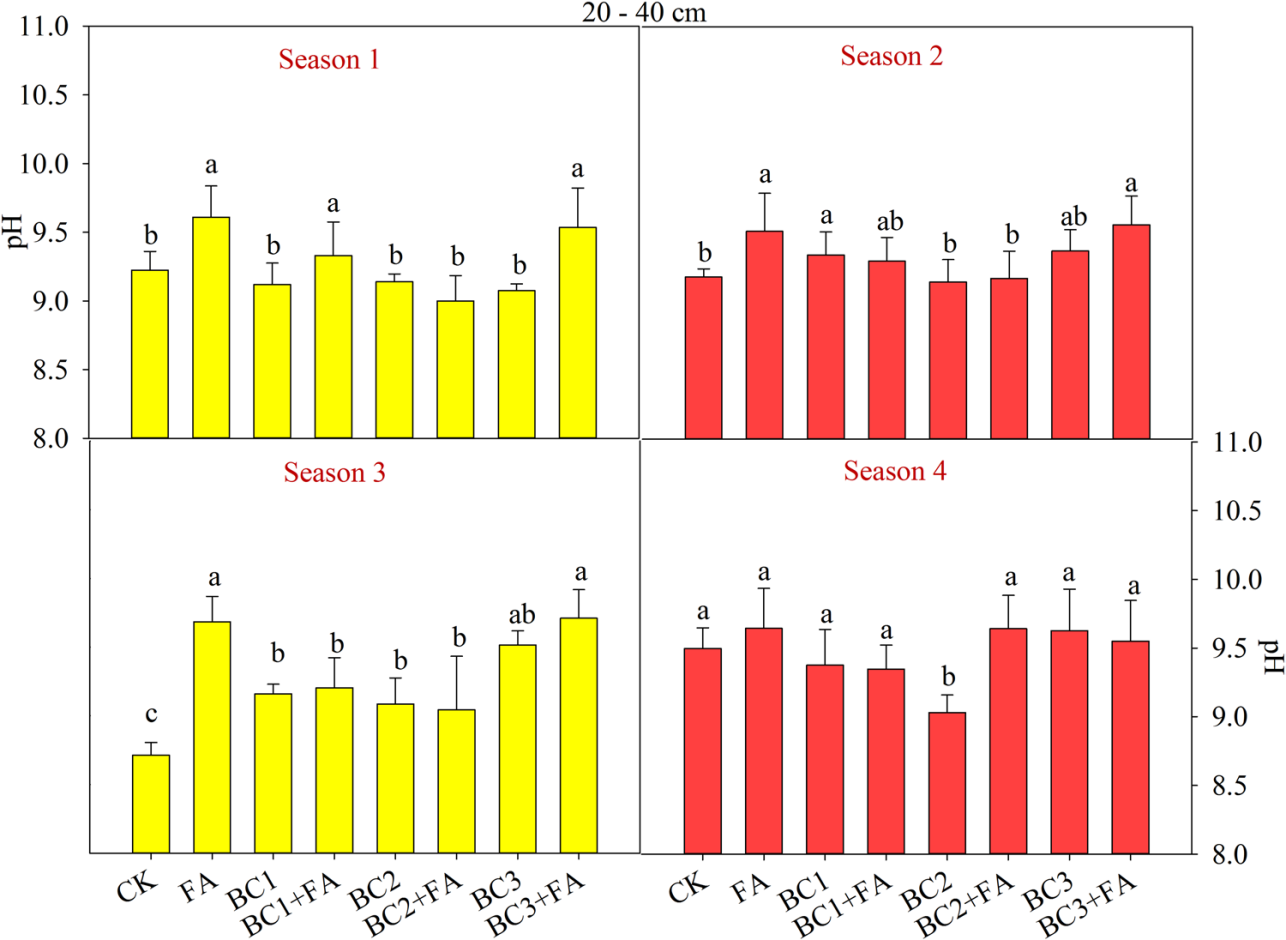
Soil pH values at 20–40 cm depth as affected by different treatments during the studied four seasons. CK is control, BC1 is 7.5 t ha^−1^ biochar, BC2 is 15 t ha^−1^ biochar, BC3 is 30 t ha^−1^ biochar, FA is 1.5 t ha^−1^ fulvic acid. Values are means ± SE (*n* = 3). For each season, bars within each panel with different letters are significantly different according to LSD at *p* < 0.05 level. Bars with the same fill color represent the same crop species during the experiment.

The pH of the two soil layers in all treatments increased in season 1 (Fig. 3, Fig. 4). pH in high depth was little than that of the lower depth among all the treatments. The pH of all treatments during the four seasons were all higher than 8.5 showing the serious situation of soil alkalinity. No significant difference were observed in 0–20 cm soil layer in season 1 and season 2. pH showed big variation among the treatments in season 3 and season 4. Compared with CK, the pH was kept at low alkali level in BC2 and BC2 + FA. The pH of BC2 and BC2 + FA in the four seasons were 8.96, 8.97, 8.74 and 8.66, and 8.84, 9.12, 8.78 and 9.13, respectively. Regardless of the effects of fertilizers, the best treatment for decreasing EC and control pH increase was BC2 + FA.

### Soluble ions and soil nutrients

In the soil solution, Na^+^ accounts the maximum amount of cation, while the maximum amount of anion was Cl^−^(Table 1). Na^+^ and Cl^−^ content was lowest in FA treatment. K^+^ and CO_3_^2−^ + HCO_3_^−^ contents in all treatments in 0–20 cm soil layer were all lower than in 20–40 cm. However, TOC, TP, Ca^2+^, Mg^2+^ and SO_4_^2−^ contents were higher in 0–20 cm layer than 20–40 cm layer in each treatment. Na^+^ and Cl^−^ were highest in CK at the two soil layers. At the 0–20 cm soil depth, TOC reached the maximum amount in BC3, followed by BC3 + FA and BC2, which increased the TOC by 84.2, 68.4 and 42.1%, respectively. FA addition decreased the positive effect of TOC improvement in BC treatment. At the 20–40 cm layer, there were no significant difference between all treatments in TOC content. TP improved with the increase of BC application rate, and the highest content was in BC3 + FA treatments, which was 701.6 mg kg^−1^, followed by BC3 treatments and BC2 + FA treatments. Though TOC and TP were highest in BC3 and BC3 + FA treatments, Ca^2+^ and Mg^2+^ were lower in the two treatments than BC2 and BC2 + FA.

**Table 1:** Soil total organic carbon (TOC, g kg^−1^), total phosphorus (TP mg kg^−1^) and major ions composition(mg kg^−1^) at different sampling depths after the three year field trial as influenced by different treatments (mean ± SE, *n* = 3).

Item^a^	TOC	TP	K^+^	Na^+^	Ca^2+^	Mg^2+^	Cl^−^	SO_4_^2−^	CO_3_^2−^ + HCO_3_^−^
	**0–20** **cm**								
CK	3.8 ± 0.2d	487.4 ± 24.5b	17.2 ± 2.3b	370 ± 35.2a	51.5 ± 22.2c	27.7 ± 4.5b	781.6 ± 58.1a	33.5 ± 7.3a	21.4 ± 3a
FA	4.1 ± 0.8c	516.8 ± 11.3b	12.4 ± 1.1b	271.3 ± 26.3b	69.2 ± 40.1b	17.6 ± 5.3c	303.7 ± 27.8e	14.3 ± 6.7b	34.6 ± 4.1a
BC1	5.1 ± 0b	334 ± 17.9c	13.2 ± 1.1b	329.9 ± 23.8a	110.4 ± 35.6a	25.3 ± 1.1b	608.7 ± 34.9b	16.7 ± 7.1b	30.5 ± 1a
BC1 + FA	4.3 ± 0.7c	424.7 ± 18.2ab	15.6 ± 2b	289 ± 17.6b	114.4 ± 17.8a	43.7 ± 3.4a	583.7 ± 46.5c	25.4 ± 3.3ab	30.5 ± 6.1a
BC2	5.4 ± 0.7b	490.2 ± 23.6b	14.4 ± 1.7b	355.1 ± 22.1a	129 ± 12.1a	37.9 ± 2.9a	699.1 ± 38.2ab	27.3 ± 1.4a	27.5 ± 4.3ab
BC2 + FA	5.2 ± 0.3b	507.2 ± 15.1b	18 ± 3.4b	333.5 ± 15.3a	107.6 ± 1.1a	32.5 ± 5.2a	525.7 ± 11.8c	20.2 ± 4.3ab	27.5 ± 4.3ab
BC3	7 ± 0.1a	542.8 ± 17.6b	20.3 ± 3.4a	305.8 ± 17ab	75.8 ± 5.5b	20.6 ± 2.2b	457.3 ± 33.6d	17 ± 6.8b	21.4 ± 4.3b
BC3 + FA	6.4 ± 0.1a	701.6 ± 29.9a	27.5 ± 3.8a	296.2 ± 24.6b	88.8 ± 19.2b	26.2 ± 2.7b	376.1 ± 38.5e	34.2 ± 2.7a	27.5 ± 2.9ab
	**20–40** **cm**								
CK	1.4 ± 0.8	366.7 ± 36.4b	30.7 ± 2.8ab	390 ± 36.3a	65.2 ± 4.5a	18.3 ± 5.7b	751.1 ± 19a	18 ± 1.9a	32.5 ± 7.7ab
FA	1.3 ± 0.2	323.7 ± 39.5b	29.1 ± 2.3b	237.7 ± 12.1b	45.9 ± 2b	24.2 ± 2.2a	232.1 ± 39.9d	13 ± 1.9a	36.6 ± 2.6a
BC1	1.6 ± 0.2	265.5 ± 14.5c	29.9 ± 3.4b	378 ± 34a	56.6 ± 2.5a	17.1 ± 8.3b	640.2 ± 48.4a	16.5 ± 2.8	35.1 ± 10.8
BC1 + FA	1.4 ± 0.3	384 ± 53b	28.3 ± 2.4b	298.6 ± 14.7b	42.9 ± 7.9b	25.8 ± 4.5a	479 ± 39.7b	17.5 ± 1.4a	30.5 ± 3b
BC2	1.8 ± 0.8	356.2 ± 14.2b	28.7 ± 5.1b	361.1 ± 13.6a	62.2 ± 6.7a	10.6 ± 1.6c	580.7 ± 60.2ab	15.4 ± 2.3a	24.4 ± 2.3b
BC2 + FA	1.7 ± 0.2	343.6 ± 28.4b	31.1 ± 1.7ab	346.7 ± 40.8a	57.6 ± 2.3a	19 ± 2.4b	474.3 ± 26.7b	24 ± 7.3a	38.1 ± 5.1a
BC3	1.3 ± 0.1	325.4 ± 21.3b	37.1 ± 1.2a	329.9 ± 40.8a	45.4 ± 4.8b	21.8 ± 6a	422.2 ± 26.2c	7.3±3.4b	42.7 ± 7.3a
BC3 + FA	1.1 ± 0.2	492.7 ± 14.4a	28.7 ± 1.7b	281.8 ± 12.1b	59.6 ± 7.9a	14.4 ± 5.2b	357 ± 41.6d	13.2±5.5a	35.1 ± 6.5a

^a^CK is control, BC1 is 7.5 t ha^−1^ biochar, BC2 is 15 t ha^−1^ biochar, BC3 is 30 t ha^−1^ biochar, FA is 1.5 t ha^−1^ fulvic acid. Mean values ± SE in the same row of each soil depth followed by the different lowercase letters indicate significantly difference using LSD test at p < 0.05 level.

### Water holding capacity

Soil bulk density (BD) value was significantly reduced by addition of BC and/or FA in season 1 (Table 2). BD values of treatment FA, BC1, BC1 + FA, BC2, BC2 + FA, BC3 and BC3 + FA were decreased by 8.1, 10.8, 2.7, 4.7, 5.4, 4.1 and 8.1%, respectively, compared to the CK. However, soil water content (WC) was only increased in treatments BC1, BC2, BC2 + FA and BC3 + FA. The WC in BD2 + FA and BD3 + FA were all approximately 24.9% in season 1, and it was the biggest value among all treatments. Besides, filed water capacity (FWC), saturated water capacity (SWC), soil porosity (SP) and capillary porosity (CP) were all increased in all treatments compared to CK in season 1. Compared with CK, the BD value was only continuously reduced in BC3 and BC3 + FA. The BD was gradually decreased as the experiment continued. Though the SWC was increased in season 4 under all treatments compared to season 1 to season 3, FWC remained a stable value in all treatments. The BD in barley cultivation season was lower than that in maize season in each treatment, but the FWC measurements showed an opposite trend. At the end of the experiment, the highest CP and capillary water content (CWC) were in BC at 15 t ha^−1^ with FA plot (BC2 + FA), and the CP and CWC were increased by 8.9 and 16.3% than no amendment-applied plot. Otherwise, there were no significant differences in other soil physical properties between the BC2, BC2 + FA, BC3 and BC3 + FA treatments.

**Table 2:** Soil bulk density (BD), water content (WD), saturated water content (SWC), field water capacity (FWC), soil porosity (SP), capillary porosity (CP), non-capillary porosity (NCP), capillary water content (CWC) at the topsoil during the studied four seasons as influenced by different treatments (mean ± SE, *n* = 3).

	Item^a^	BD (g cm^−3^)	WC (%)	SWC (%)	FWC (%)	SP (%)	CP (%)	NCP (%)
Season 1	CK	1.48 ± 0.05a	20.69 ± 3.09ab	23.64 ± 3.91b	22.58 ± 3.63b	43.97 ± 1.94b	31.87 ± 4.49b	12.1 ± 3.86b
	FA	1.36 ± 0.27b	19.52 ± 4.02b	27.26 ± 1.13a	25.66 ± 1.03ab	48.48 ± 1.22a	34.82 ± 7.17a	13.66 ± 1.04ab
	BC1	1.32 ± 0.05b	24.67 ± 0.93a	31.91 ± 2.05a	28.74 ± 0.31a	50.53 ± 1.87a	31.75 ± 7.35b	18.78 ± 5.48a
	BC1 + FA	1.44 ± 0.03a	19.78 ± 3.66b	24.58 ± 1.82b	22.95 ± 1.41b	45.8 ± 1.13ab	35.74 ± 4.25a	10.06 ± 2.28b
	BC2	1.41 ± 0.02a	22.48 ± 1.59ab	26.24 ± 0.48a	24.17 ± 0.9ab	46.95 ± 0.01a	32.05 ± 1.46b	14.91 ± 1.45ab
	BC2 + FA	1.4 ± 0.02a	24.89 ± 2.84a	28.145 ± 1.77a	27.02 ± 2.18a	47.28 ± 0.93a	36.25 ± 4.48a	11.03 ± 3.55b
	BC3	1.42 ± 0.16a	19.62 ± 3.59b	25.645 ± 6.31a	23.26 ± 4.99ab	46.32 ± 5.76a	34.81 ± 6.51ab	11.52 ± 0.76b
	BC3 + FA	1.36 ± 0.01b	24.99 ± 2.55a	29.085 ± 1.46a	27.55 ± 1.44a	48.78 ± 0.21a	37.81 ± 1.22a	10.97 ± 1b
Season 2	CK	1.35 ± 0.04a	29.35 ± 1.11a	34.37 ± 2.75ab	32.6 ± 1.42b	49 ± 1.52ab	44.03 ± 0.62a	4.98 ± 0.9b
	FA	1.36 ± 0.03a	28.85 ± 1.66b	33.28 ± 1.5ab	32.26 ± 1.5b	48.66 ± 1.2b	43.86 ± 1.13a	4.8 ± 0.68b
	BC1	1.34 ± 0.02a	28.65 ± 2.09b	34.69 ± 0.89ab	33.35 ± 0.65b	49.51 ± 0.7ab	44.61 ± 0.25a	4.89 ± 0.46b
	BC1 + FA	1.32 ± 0a	30.28 ± 0.28a	35.95 ± 0.94ab	33.94 ± 0.07b	50.37 ± 0.02a	44.64 ± 0.1a	5.74 ± 0.12b
	BC2	1.32 ± 0.02a	29.23 ± 0.73a	39.95 ± 1.64a	36.71 ± 0.49a	53.98 ± 0.82a	44.77 ± 0.21a	9.22 ± 1.02a
	BC2 + FA	1.33 ± 0.03a	29.3 ± 1.66a	34 ± 2.13ab	32.39 ± 0.96b	49.69 ± 0.97ab	43.17 ± 0.45a	6.53 ± 0.52b
	BC3	1.28 ± 0.05b	31.34 ± 0.85a	38.33 ± 1.96a	37.02 ± 2.44a	51.67 ± 1.87a	47.35 ± 1.29a	4.32 ± 0.58b
	BC3 + FA	1.27 ± 0.06b	30.68 ± 1.92a	30.73 ± 6.63b	35.83 ± 2.3a	52.22 ± 2.3a	45.3 ± 0.73a	6.93 ± 1.57b
	Item	BD (g cm^−3^)	WC (%)	SWC (%)	FWC (%)	SP (%)	CP (%)	NCP (%)
Season 3	CK	1.38 ± 0.01a	24.92 ± 6.83a	28.59 ± 4.49a	23.2 ± 6.07a	47.8 ± 0.2a	38.11 ± 6.89ab	9.69 ± 6.69ab
	FA	1.32 ± 0.11b	25.95 ± 6.92a	31.23 ± 8.63a	23.94 ± 6.86a	50.15 ± 4.12a	39.14 ± 6.89a	11 ± 2.97a
	BC1	1.4 ± 0.12a	26.76 ± 2.06a	29.38 ± 11.31a	23.83 ± 9.89a	47.28 ± 4.52a	39.78 ± 12.64a	7.5 ± 8.11b
	BC1 + FA	1.36 ± 0.1a	24.41 ± 6.41a	28.92 ± 6.62a	22.59 ± 5.7a	48.63 ± 3.74a	37.75 ± 6.77b	10.88 ± 3.03a
	BC2	1.38 ± 0.08a	24.71 ± 7.04a	28.31 ± 7.06a	22.62 ± 6.37a	47.74 ± 2.91a	37.67 ± 7.28b	10.07 ± 4.37a
	BC2 + FA	1.38 ± 0.01a	23.91 ± 0.58a	28.29 ± 1.16a	22.28 ± 1a	47.94 ± 0.22a	37.65 ± 1.39b	10.29 ± 1.17a
	BC3	1.31 ± 0.04b	25.39 ± 5.18a	30.76 ± 4.5aa	23.87 ± 5.09a	50.51 ± 1.43a	40.21 ± 4.38a	10.3 ± 2.95a
	BC3 + FA	1.36 ± 0.1ab	24.96 ± 6.49a	30.04 ± 7.6aa	22.96 ± 6.53a	48.69 ± 3.84a	38.26 ± 5.78ab	10.43 ± 1.94a
Season 4	CK	1.31 ± 0.28a	12.52 ± 1.04a	37.21 ± 2.4b	27.06 ± 1.35ab	50.68 ± 1.62a	42.17 ± 4.09a	8.5 ± 5.59c
	FA	1.32 ± 0.05a	14.26 ± 1.05a	37.74 ± 1.8b	25.97 ± 1.22b	51.45 ± 2.8b	44.18 ± 9.19a	7.27 ± 8.36c
	BC1	1.26 ± 0.24ab	10.76 ± 2.34b	40.8 ± 0ab	26.72 ± 0.12ab	52.39 ± 1.51ab	36.06 ± 2.1b	16.33 ± 0.59a
	BC1 + FA	1.19 ± 0.88c	13.05 ± 1.08a	44.13 ± 5.04a	30.31 ± 3.36a	55.15 ± 2.91a	42.04 ± 3.34a	13.11 ± 4.39b
	BC2	1.25 ± 0.21ab	12.97 ± 2.28a	40.9 ± 0ab	30.87 ± 0.21a	52.91 ± 0.08ab	39.06 ± 0.55ab	13.85 ± 0.63b
	BC2 + FA	1.23 ± 0.03b	13.11 ± 1.03a	42.1 ± 1.42a	27.5 ± 3.02ab	53.75 ± 0.01a	45.94 ± 2.08a	7.81 ± 2.09c
	BC3	1.22 ± 0.32b	13.25 ± 1.85a	41.7 ± 0ab	27.77 ± 0.34ab	53.86 ± 0.07a	34.74 ± 0.65b	19.12 ± 0.57a
	BC3 + FA	1.25 ± 0.11ab	12.73 ± 2.05a	40.79 ± 1.44ab	27.68 ± 0.05ab	52.98 ± 1.82ab	44.11 ± 1.33a	8.87 ± 3.15c

^a^CK is control, BC1 is 7.5 t ha^−1^ biochar, BC2 is 15 t ha^−1^ biochar, BC3 is 30 t ha^−1^ biochar, FA is 1.5 t ha^−1^ fulvic acid. Mean values ± SE in the same row of each studied season followed by the different lowercase letters indicate significantly difference using LSD test at p < 0.05 level.

### Crops yield

**Figure 5:**
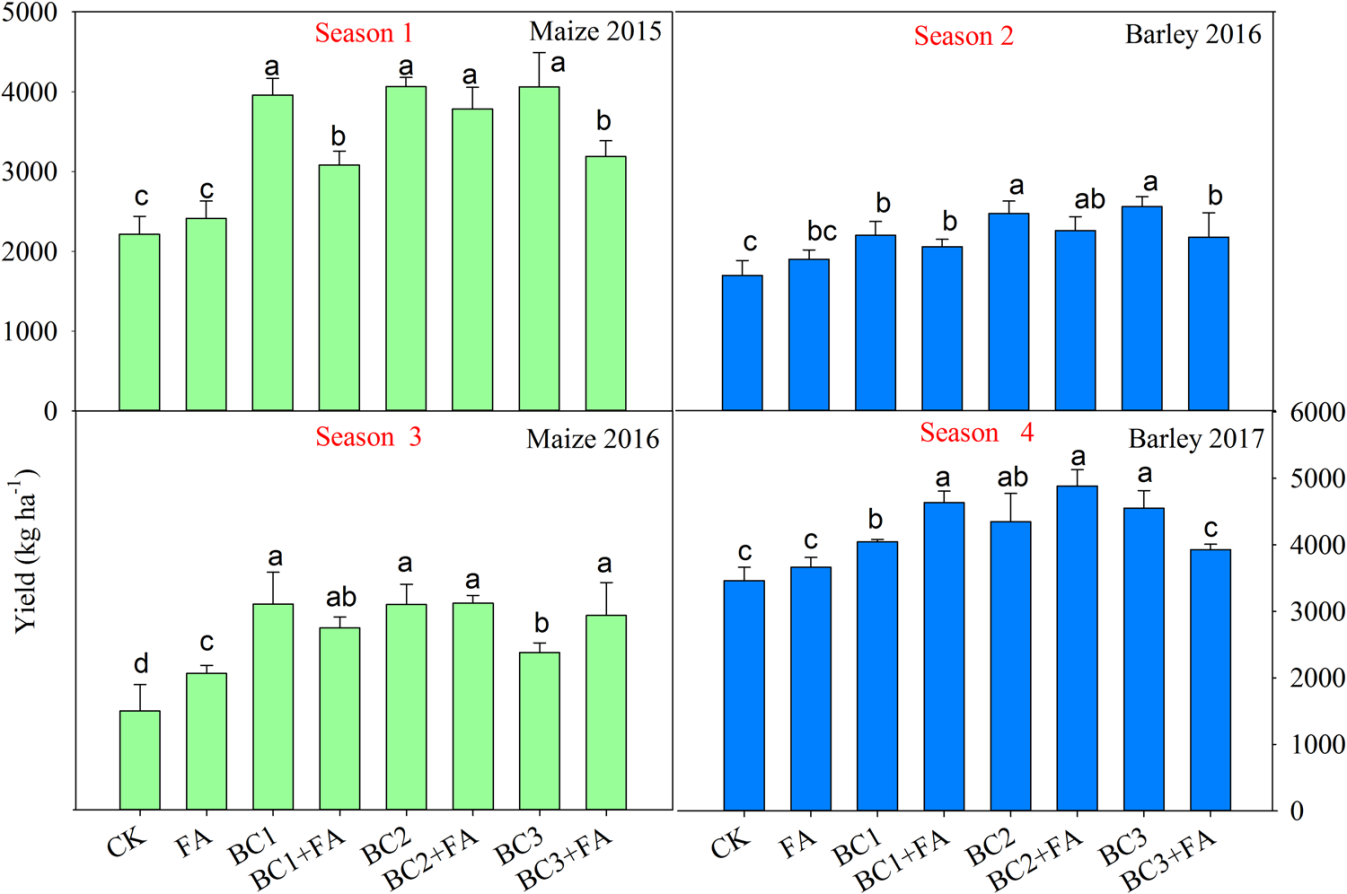
Grain yield of each season as influenced by different treatments during the three year trial. CK is control, BC1 is 7.5 t ha^−1^ biochar, BC2 is 15 t ha^−1^ biochar, BC3 is 30 t ha^−1^ biochar, FA is 1.5 t ha^−1^ fulvic acid. Values are means ± SE (*n* = 3). For each season, bars within each panel with different letters are significantly different according to LSD at *p* < 0.05 level. Bars with the same fill color represent the same crop species during the experiment.

**Figure 6:**
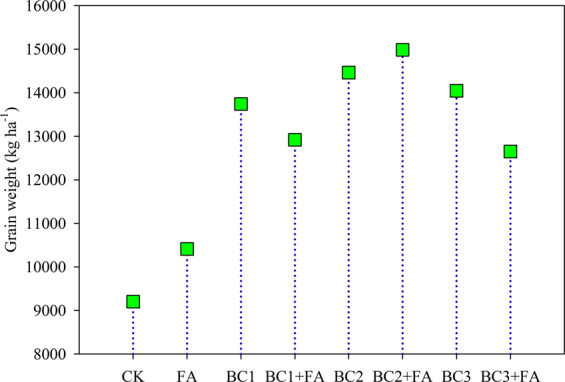
Total grain yield of the four studied seasons as influenced by different treatments. CK is control, BC1 is 7.5 t ha^−1^ biochar, BC2 is 15 t ha^−1^ biochar, BC3 is 30 t ha^−1^ biochar, FA is 1.5 t ha^−1^ fulvic acid. Values are means ± SE (*n* = 3). For each season, bars within each panel with different letters are significantly different according to LSD at *p* < 0.05 level.

During the 4 growing seasons, the grain yield of summer maize and winter barley, in general, had an increasing tendency with biochar and fulvic acid addition. Data of grain yields in each season and total yields of the four seasons are shown in (Figs. 5, 6). Remarkably, significant increases were observed in maize yield in season 1 by 78.7, 39.2, 83.4, 70.8, 83.3 and 43.9%, respectively, with BC1, BC1 + FA, BC2, BC2 + FA, BC3 and BC3 + FA over the control. Although the barley yields in season 2 were all lower than in other season, BC in combination with FA significantly improved yield. The highest yield in the season 1 and season 2 was observed in the BC3 treatment, which was 4063.6 and 3069.3 kg ha^−1^, respectively. In season 3 and season 4, a significant increase in grain yield was obtained in 15 t ha^−1^ BC + FA treatment. The yield in the BC2 + FA was 3108.9 and 4884.7 kg ha^−1^ in season 3 and season 4, respectively. FA added alone had no significant effect on crop yield measured through the four seasons. In contrast, the application of BC in combination with FA had a significant effect on yield improvement. Although the grain yield was variable among the four growing seasons, BC and FA addition caused little fluctuation. As a result, the total yield during the four seasons was statistically different. The highest total grain yield was seen in the BC2 + FA treatment, and the total yield was increased by 62.9% over the CK. The FA + BC1 or BC3 treatment showed a lower total yield than that of BC1 or BC3 treatment (Fig. 6).

## Discussion

Salt stress is a major abiotic stress on crops in coastal land because of its direct impact on seed germination, growth and finally grain yield^33^. Although some plants develop several mechanisms to cope up salinity stress like ion regulation by Na/H antiporter; synthesis of amino acids like proline, aspartic acid and valine; etc.^34^, many food crops are unable to adapt to the saline environment. In China, food demand is a rigid problem, the essence of the problem of grain self-sufficient rate is how to increase crop production. But due to the lack of water in saline-alkali lands, dry farming is becoming the most important way in water saving cultivation. Many practices have been conducted to improve crop yields in saline soil, and the two major methods are breed new salt-tolerant variety by agricultural biotechnology and soil remediation. Soil remediation is an effective way, and it costs shorter time than agricultural biotechnology^28^. There are many researches on the effect of biochar and humic acid application on saline soil improvement^29,35–37^. However, it is difficult to compare the different studies, as each experiment differs in terms of various produce method, original material, application usage, soil and management^38^. The results here showed that the amendment of BC and FA caused a significant decrease in salt stress to, and thus improvement of, maize and barley crops.

**Figure 7:**
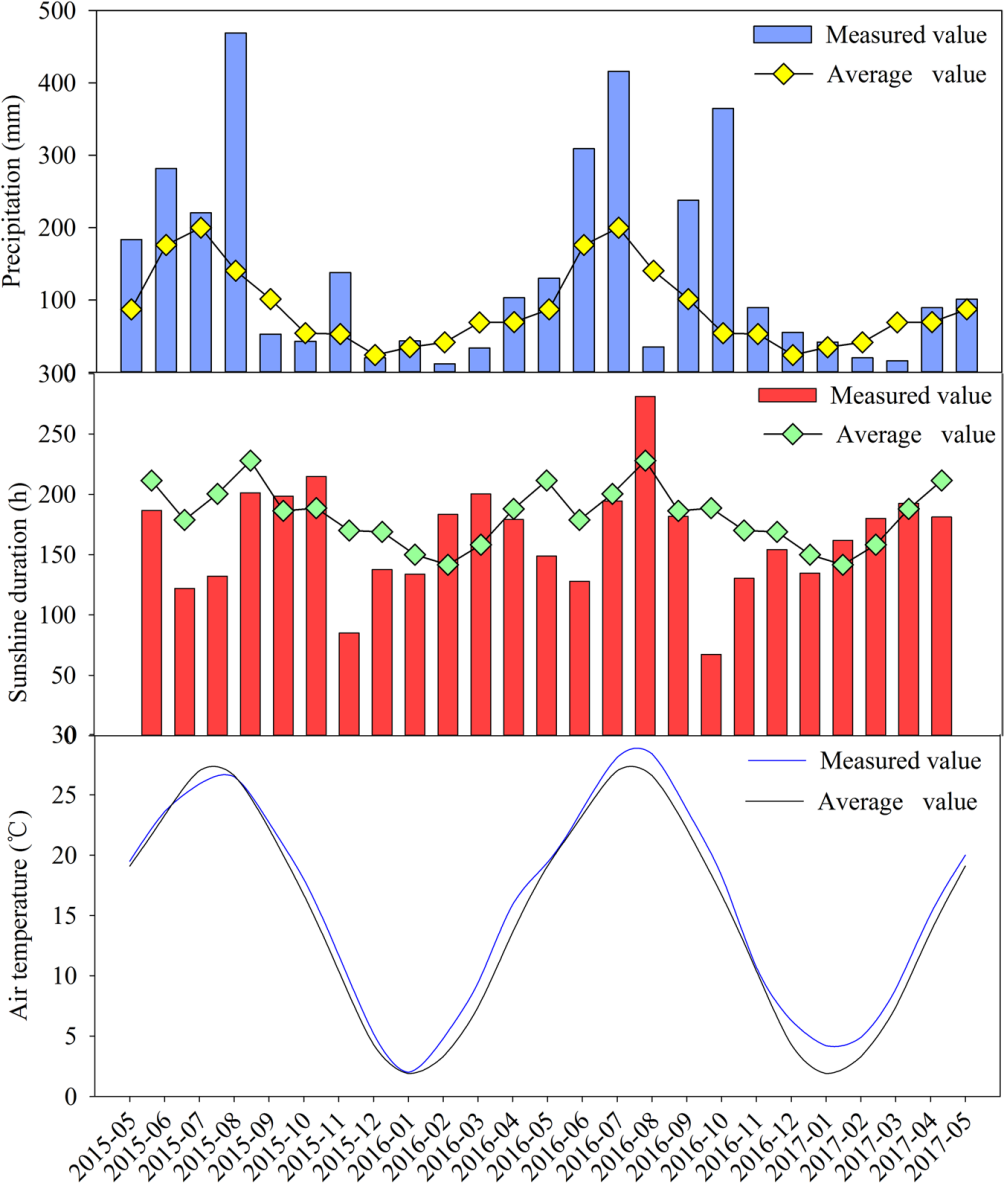
Month average precipitation, month sunshine duration and month average temperature during May 2015 to May 2017.

Though the EC at depth of 0–20 cm was significantly decreased in FA treatment, its EC values in 20–40 cm soil layer was higher than that of BC2 + FA, BC3 and BC3 + FA treatments. BC can be used as an ameliorant in salt affected soils to reduce salinity and alkalinity stress by adsorption of salt^19,39^. In this study, BC was mainly broadcasted in 0–20 cm soil layer, and the EC of BC addition treatments at depth of 0–20 cm was higher than that of 20–40 cm soil layer. Otherwise, treatments contain BC had higher EC values than FA treated plots during the four growing seasons. The variation of EC values of the four seasons was very significant, this may be caused by the great quantity of rain during the period of maize cultivation (Fig. 7). The precipitation volume was more than double the mean precipitation content of the past 20 years during the four seasons in present study. Due to irregular evaporation and precipitation around the year, soil salts migrate to the soil surface in dry season and leached down to the subsoil by the rain in the rainy season^4^. The sunshine duration decreased gradually with the increased air temperature during the experiment proved the global warming. Jenkinson *et al*.^40^ had reported that one effect of global warming will be to accelerate the decomposition of soil organic carbon, thereby releasing CO_2_ to the atmosphere, which will further accelerate the warming period.

BC and FA addition improved soil pH at depth of 0–40 cm (Figs. 3, 4). The increase of soil pH under natural cultivation condition has been reported in^4,41^. In coastal lands, there is excessive Na^+^ and a certain amount of CO_3_^2−^ + HCO_3_^−^ in soil. The frequent alternation of shine and rainy weather accelerated the frequency of salts contacting with the soil colloid, which accelerate the soil alkalization^42^. High soil pH influences chemical form of elements, and it can increase or decrease availability and uptake of nutrition. Although increased pH decreases the uptake of Ca^2+^ and Mg^2+^, it can promotes the release of colloid-adsorbed Si to the soil solution and increase crop weight at high pH (9.5)^43^.

Coastal saline soils contain a lot of chlorides in which chloride and sodium account for 60–80% of the anion and cation concentrations, respectively^44^. Salt stress influences crops growth and development through osmotic stress and ion toxic. BC + FA treatments reduced the Na^+^, Cl^−^, SO_4_^2−^ contents and increased K^+^, Ca^2+^, Mg^2+^ contents at depth of 0–20 cm after the four growing seasons. Biochar altered negative consequences of salinity by reducing Na+ uptake or by eliminating Na^+^ from the root cells, and it released K^+^, Ca^2+^, Mg^2+^ in soil which is beneficial for crop growth^45^. The presence of various functional groups in BC and FA makes them a suitable choice for the adsorption of various salts present in the soils, thus mitigating the salinity of soil^46^.

Setia *et al*.^47^ reported that soil salinity decrease global soil organic carbon stocks and their newly modelling suggested that world soils may lose 6.8 Pg SOC due to salinity by the year 2100. BC consists of a large degree of recalcitrant carbon, which could remain in soil for more than 100 years, and thus, biochar could be very useful in fixing the carbon in the soil^48^. FA consists of macromolecular complex comprised of aromatic hydroxyl carboxylic acid which is easy for plant or microorganisms to absorb. BC and FA improved the soil organic carbon significantly at the depth of 0–20 cm soil layer after the four growing seasons (Table 1), and the TOC were not affected by the BC and FA application in 20–40 cm soil. TOC in BC + FA was lower than that of BC indicating that FA accelerated the BC decomposition. This effect may be caused by the stimulation function of FA on microbial activities^49,50^. In good agreement with Subedi *et al*.^51^, who had reported that biochar amendments increased soil phosphorus availability for low fertility soils, the total phosphorus content in the soil at depth of 0–20 cm here increased from 487.4 mg kg^−1^ in the CK up to 701 mg kg^−1^ for BC3 + FA at the last experimental season. High BC application rate showed a higher ability on maintaining soil nutrition for its highly porous structure and the large surface area. Xu *et al*.^52^ reported that the effect of BC on plant biomass was equal to phosphorus fertilization in first growing season. So the BC and FA can also release nutrients to soil which improved plant growth.

Soil BD and water holding capacity are essential properties representing the soil physical condition. The application of BC or BC + FA decreased the soil BD in topsoil (Table 2) during the four seasons. The BD in each treatment in maize cultivation was higher than that of barley cultivation. This might be attributed to the extreme precipitation leading to the soil compaction during the maize cultivation^53^. The water holding capacity is affected by many factors, such as bulk density, clay particle content, humic matter, porosity^54^. The BC + FA amendments not only increased water holding capacity but also optimized soil porosity composition. Application of BC and FA accommodated WC, SWC, FWC and CWC values to the optimal ranges and increased water retention capacity. Similar results were reported in^25^, which reported that biochar and humic acid improved the particle structure of the growth substrate. BC2 + FA had the highest CP and CWC values in season 4. BC + FA treatments showed a higher CP than BC alone. This might be primarily due to the reduction of soil BD, higher small porosity and aggregation stability^55^. Furthermore, biochar and fulvic acid has large surface energy which is helpful for improving the water holding capacity.

Improved crop yields with BC and/or FA addition in greenhouse^25,56^ as well as field studies^7,19^ were reported for the well germination of seeds and well growth till maturing. In the first two seasons, the most crop yields were obtained in BC2 and BC3 treatments. The improvable effects of BC on grain yield improvement was higher than that of BC + FA. But in the last two seasons, BC2 + FA amendment showed a highest production. The maximum total yields of the four growing seasons was obtained in BC2 + FA. FA application rate was consistently the same, but it slightly increased the crop yield during the four seasons compared with CK. So BC applied with appropriate rate in combination with FA was a good way to increase crop yields^5^.

## Materials and methods

### Experimental site

The experiment was carried out during the period of May 2015 to May 2017 at Huanghai Raw Seed Farm (32°38′, 120°52′), in Dongtai City, Jiangsu Province, China. This site is typical landscape of the coastal saline agriculture in the subtropical area of East China. The average annual precipitation is 1042 mm, and with the evaporation of 1417 mm. However, almost 70% of annual rainfall appeared in rainy season, which is from June to September^57^. The average annual temperature, wind velocity and relative humidity are 14.6 °C, 3.3 m s^−1^ and 81% respectively. The site is in a subtropical area, characterized by a Northwest monsoon in winter and a Southeast monsoon from spring to autumn.

The experimental site was approximately 2.5 km to the coastline of China Yellow Sea. The farm was enclosed and reclaimed from coastal mudflats in 2005.The soils were formed from fluvial and marine deposits, and the predominant soil type is silt loam, and it can be classified as a loamy, Aquic Halaquepts according to *Soil Taxonomy* (Soil Survey Staff, US Department of Agriculture, 2014). Dry farming is the predominant cultivation method for the shortage of water. In this area, the common summer crops are soybean (*Glycine max L*.) and maize (*Zea mays L*.), and the conventional winter crops are barley (*Hordeum vulgare L*.), wheat (*Triticum aestivum L*.) and rape (*Brassica napus L*.). Maize-barley rotation is the traditional farming method in this region. However, crop yields are far below the potentially attainable levels, due to multiple limiting factors. These factors contained high soil salinity, seawater intrusion to groundwater, low organic matter content in soil and lack of effective amelioration method. Chemical fertilizers were normally used for improving yields, but its utilization efficiency was very low in the salt-affected soil. The major soil characteristics (measured at the start of the experiment) are listed in Table 3.

**Table 3:** Basic chemical and physical properties of the soil (0–20 cm), biochar, and fulvic acid.

Soila		Biochar		Fulvic acid	
EC (μS cm^−1^)	680.00	Feedstock	wheat straw	Pure substance (%)	99.80
pH (water 1: 5)	9.24	Pyrolysis temperature (°C)	350–550	pH (water 1: 5)	4.52
TOC (g kg^−1^)	3.55	EC (μS/cm)	820.00	C (%)	55.90
Bulk density (g cm^−3^)	1.38	pH (water 1: 10)	10.4	H (%)	2.35
Total N (g kg^−1^)	0.28	TOC (g/kg)	467	N (%)	0.76
Sand (%)	3.48	Bulk density (g/cm^3^)	0.65	S (%)	2.18
Silt (%)	75.76	N (%)	5.90	O (%)	38.81
Clay (%)	20.76	Total ash (%)	20.8	H/C (%)	0.50
Na^+^ (mg kg^−1^)	400	Ca (mg/kg)	10.10	O/C (%)	0.52
Cl^−^ (mg kg^−1^)	550	Mg (mg/kg)	6.09	N/C (%)	0.01
CEC (cmol kg^−1^)	2.42	CEC (cmol/kg)	35.15	Acidic groups (mmol g^−1^)	9.39

^a^EC is electrical conductivity, TOC is total organic carbon, CEC is cation exchange capacity.

### Biochar and fulvic acid

The commercially available biochar (BC) was derived from mechanically chipped wheat straw pyrolysed at 350–550 °C in a vertical kiln from the Shangqiu Sanli New Energy Co., Ltd (Henan Province, China). Biochar was ground to pass a 2 mm sieve before its application to the soils. Fulvic acid (FA) was produced by airslake coal from Pingxiang Red Land Humic Acid Co. Ltd. The physiochemical properties of the biochar and fulvic acid are shown in Table 3.

### Field experimental design

The fields were cropped with rotations of maize and barley or wheat before sowing. The field experiment was conducted on fields with a flat or gently sloping topography. In the first year, 24 blocks were marked in size of 6 m×8 m, and the spacing distance of each block was 35 cm. The following eight treatments were arranged: CK: served as the control without biochar or fulvic acid; FA: each plot contained 1.5 t ha^−1^ fulvic acid; BC1: each plot contained 7.5 t ha^−1^ biochar; BC2: each plot contained 15 t ha^−1^ biochar; BC3:each plot contained 30 t ha^−1^ biochar; BC1 + FA, BC2 + FA and BC3 + FA. For the experiment, all treatments were performed in a completely randomized design with three replications. During the first cultivation season, all of the treatments of the field experiment were arranged as follows. Biochar or FA was broadcasted on the soil surface and mixed with the topsoil by machinery plowing to a depth of 20 cm. In May 2015, maize (*Zea mays* L.) was sown after the treatments application following standard farming practices. The plots in the following three cultivation season were amended no more biochar but only FA in the same dose. The summary of important field managements and sampling dates are elaborated in Table 4. The experiment lasted from 2015 to 2017.

**Table 4:** Summary of important field management and sampling dates.

Year	Crop	Variety	Planting date	Fertilization date	Harvest date	Soil sampling date
2015	Maize	Suyu 30	30 May	28 May	1 Oct.	30 Sept.
(season 1)				20 Jun.		
				2 Aug.		
2015/2016	Barley	Supi 4	10 Nov.	10 Nov.	10 May	29 April
(season 2)				5 Mar.		
2016	Maize	Suyu 30	10 Jun.	9 Jun.	7 Oct.	1 Oct.
(season 3)				11 Jul.		
				15 Aug.		
2016/2017	Barley	Supi 4	15 Nov.	15 Nov.	20 May	19 May
(season 4)				10 Mar.		

### Soil sampling and measurements

Soil samples were collected from 3 plots from each treatment in mature stage of the four season (Table 4). Soils at depth of 0–20 cm and 20–40 cm were taken separately at the same time. Undisturbed soil cores were taken from soil at depth of 10–15 cm by a cylinder of 100 cm^3^ in volume to determine bulk density (BD) and water holding capacity. Three replicate samples were taken and bulked in a plastic bag from each plot. The soil samples were air-dried in shade situation for subsequent measurements. The air-dried soils collected from the four season cultivation were sieved to pass 2 mm for determining soil salinity (measured as electrical conductivity, EC), pH in 1:5 *w/v* ratio soil suspensions. Otherwise, the soil samples obtained from the last season were sieved to pass 1 mm for measuring soluble ions composition (Na^+^, K^+^, Ca^2+^, Mg^2+^, Cl^−^, CO_3_^2−^, HCO_3_^−^, and SO_4_^2−^). Soil total organic carbon and prosperous content were tested while the soils were sieved to pass 0.25 mm. Soil properties were analyzed following the standard protocol (Bao, 2000).Soil EC and pH were determined in distilled water at a ratio of 1:5 *w/v* by mechanical conductivity and pH sensors (SevenExcellence Cond meter, Mettler Toledo, CH). TOC analyses followed the oxidation method with potassium dichromate. Total phosphorus content (TP) was measured using an auto discrete analyzer (CleverChem, Germany). For analysis of Ca^2+^, Mg^2+^, Cl^−^, CO_3_^2−^, HCO_3_^−^, and SO_4_^2−^, a potentiometric titration (T70, Teller Toledo, CH) was used. Na^+^ and K^+^ were measured with flame photometry (FP6400, JingKe, China).

### Yield measurements

When maize and barley crops started senescing after reaching physiological maturity, all crops in each plot were harvested. Yield of maize/ barley grain per hectare was calculated by the weight of dried grains of each treatment. After the grains all collected, the aboveground crop residues were removed from the field plots.

### Statistical analysis

Statistical analyses and graphical design was carried out using SPSS 19.0 software (SPSS Inc., Chicago, IL, USA) and SigmPlot 12.5 (Systat Software Inc., California, USA). All results were expressed as means and standard deviations (SD). Significance for differences between the treatment means was calculated by one-way analysis of variance (ANOVA), with probability defined at 0.05.

## Conclusions

The current study demonstrated that combined amendment of BC and FA significantly improved both the physical and chemical conditions of the salt-affected soil and thus increasing crop yields in a maize-barley rotation system. This could be attributed to the multiple benefits on salt reduction, water retention, nutrient supply and crop growth improvement. Compared to the control, amendment with 15 t ha^−1^ biochar and 1.5 t ha^−1^ fulvic acid results in the largest changes of the total grain yields of the four experimental seasons. Considering that the crop yields has a strong relation with soil fertility and microbial activities, further investigation is required to evaluate the impact of BC and FA on soil nutrition and microbial community. Although many factors such as weeds, pests and timing of field operations may have an effect on yield, the trial allowed us to identify various ways in which BC and FA could have affected yields. In summary, biochar derived from wheat straw in combination with fulvic acid can reduce the secondary salinization risk in soil and help to improve productivity and crop yields.
